# Cooperation between MEF2 and PPARγ in human intestinal β,β-carotene 15,15'-monooxygenase gene expression

**DOI:** 10.1186/1471-2199-7-7

**Published:** 2006-02-21

**Authors:** Xiaoming Gong, Shu-Whei Tsai, Bingfang Yan, Lewis P Rubin

**Affiliations:** 1 Department of Pediatrics, Women and Infants' Hospital of Rhode Island and Brown Medical School, Providence, Rhode Island, USA; 2 Program in Fetal Medicine, Women and Infants' Hospital of Rhode Island and Brown Medical School, Providence, Rhode Island, USA; 3 Department of Biomedical and Pharmaceutical Sciences, University of Rhode Island, Kingston, Rhode Island, USA; 4 Department of Cell Biology, Lerner Research Institute, The Cleveland Clinic Foundation, Cleveland, Ohio, USA

## Abstract

**Background:**

Vitamin A and its derivatives, the retinoids, are essential for normal embryonic development and maintenance of cell differentiation. β, β-carotene 15,15'-monooxygenase 1 (BCMO1) catalyzes the central cleavage of β-carotene to all-*trans *retinal and is the key enzyme in the intestinal metabolism of carotenes to vitamin A. However, human and various rodent species show markedly different efficiencies in intestinal BCMO1-mediated carotene to retinoid conversion. The aim of this study is to identify potentially human-specific regulatory control mechanisms of BCMO1 gene expression.

**Results:**

We identified and functionally characterized the human BCMO1 promoter sequence and determined the transcriptional regulation of the BCMO1 gene in a BCMO1 expressing human intestinal cell line, TC-7. Several functional transcription factor-binding sites were identified in the human promoter that are absent in the mouse BCMO1 promoter. We demonstrate that the proximal promoter sequence, nt -190 to +35, confers basal transcriptional activity of the human BCMO1 gene. Site-directed mutagenesis of the myocyte enhancer factor 2 (MEF2) and peroxisome proliferator-activated receptor (PPAR) binding elements resulted in decreased basal promoter activity. Mutation of both promoter elements abrogated the expression of intestinal cell BCMO1. Electrophoretic mobility shift and supershift assays and transcription factor co-expression in TC-7 cells showed MEF2C and PPARγ bind to their respective DNA elements and synergistically transactivate BCMO1 expression.

**Conclusion:**

We demonstrate that human intestinal cell BCMO1 expression is dependent on the functional cooperation between PPARγ and MEF2 isoforms. The findings suggest that the interaction between MEF2 and PPAR factors may provide a molecular basis for interspecies differences in the transcriptional regulation of the BCMO1 gene.

## Background

Vitamin A, an essential micronutrient, is required for embryonic development and pattern formation, postnatal growth, reproduction, epithelial maintenance, immunity and vision [1-5]. With the exception of the retina, where 11-*cis*-retinal acts as the chromophore for rhodopsin [6], biological activities of vitamin A are largely mediated by isomers of retinoic acid (RA). RAs bind members of the RA receptor (RAR) and retinoid X receptor (RXR) families of ligand-dependent transcription factors to regulate transcriptional rates of retinoid response genes. Vitamin A deficiency is associated with histological abnormalities in epithelial tissues [7], decreased host resistance to tumor cells and infectious organisms [8], and increased susceptibility to environmental carcinogens [9].

Animals, incapable of synthesizing vitamin A *de novo *from isoprenoid precursors, require dietary intake of preformed vitamin A, largely as retinyl esters, or must derive retinoids from metabolism of plant β-carotene and related carotenoids. In humans, provitamin A carotenoids contribute 40–80% of total vitamin A stores [10]. Conversion of β-carotene to vitamin A is catalyzed by the enzyme β, β-carotene 15,15'-monooxygenase (E.C. 1.13.11.21., BCMO1) [11,12]. The structurally related β-carotene 9',10'-oxygenase (BCMO2) catalyzes the quantitatively minor eccentric cleavage of β-carotene to β-apo-10'-carotenal, retinol and β-ionone [13,14].

In rodents, nearly all absorbed β-carotene is converted to retinol in the intestine [15] except at very high doses [16]. In contrast, humans convert only a portion of ingested β-carotene to vitamin A so that up to 15–30% of absorbed β-carotene remains intact [17-19] and is delivered to tissues. Several lines of evidence suggest that intestinal BCMO1 activity is subject to transcriptional regulation [20,21]. However, the mechanisms governing species-specific differences in efficiency of dietary β-carotene to retinoid cleavage remain unclear.

The human BCMO1 cDNA encodes a 63-kDa protein with homologies to members of a large and diverse family of polyene chain oxidases and carotenoid cleavage enzymes [22,23]. Although BCMO1 can be detected in several tissues, its expression is most pronounced in intestinal mucosa and liver [24]. BCMO1 expression is downregulated in rat intestine by β-carotene and RA [20]. In addition, recent data demonstrated that peroxisome proliferator-activated receptor γ (PPARγ) regulates transcription of the mouse BCMO1 gene [21]. The human BCMO1 promoter sequences required for regulation of BCMO1 gene expression have not previously been defined.

We have isolated and characterized the human BCMO1 promoter region and identified several functional *cis*-acting elements. We report that in the human, unlike murids, myocyte enhancer factor 2 (MEF2) and PPAR transcription factors interactively regulate intestinal cell BCMO1 gene expression. These data suggest that cooperation between MEF2 and PPAR factors may provide a molecular basis for the species differences between rodent and human in the transcriptional regulation of BCMO1 gene.

## Results

### Identification of *cis*-acting elements in the BCMO1 promoter

Computational analysis of putative *cis-*acting elements located within the ~1.0 kb human BCMO1 5'-flanking sequence was performed using the TRANSFAC database [25] and TESS program. Several potential *cis-*acting elements were identified including, but not limited to, putative binding sites for CRE, RAR, MEF2, C/EBP, IRF1, GATA1, AP2 and PPAR, and a TATA box (Additional File 1, A). Since the mouse BCMO1 gene is regulated by PPARγ [21], initially we examined functionality of the PPAR binding sequence in the human promoter. Comparison between the human and mouse BCMO1 ~1.0 kb of genomic 5'-flanking sequences revealed approximately 33% homology with considerable gaps (Additional file 1, B). Several potential protein-DNA binding sites including those for IRF1, GATA1, AP2, PPAR and TATA are present in the same sequential order in the human and mouse BCMO1 promoters.

### Functional analysis of human BCMO1 basal promoter activity in TC-7 cells

**Figure 1:**
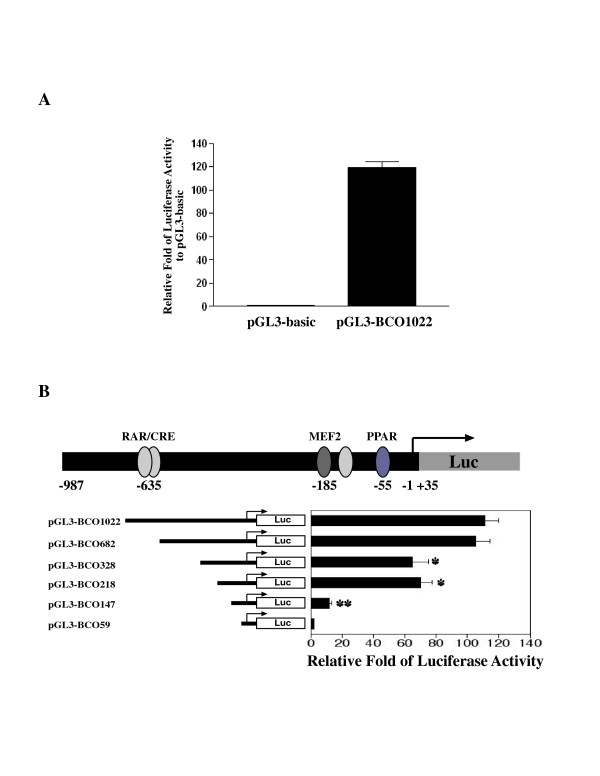
**Mapping of the regulatory elements in the BCMO1 promoter in TC-7 cells. A. **Transactivating activity of human BCMO1 promoter. A 1022-bp fragment was used for the construction of the BCMO1 reporter plasmid, pGL3-BCO1022, as described in "Methods". Luciferase activity was measured in total cell extracts obtained from TC-7 cells 24 h after transient transfection and compared with cellular luciferase activity for transfection of the control empty vector, pGL3-basic. **B. ***Above*, a schematic diagram of the BCMO1 promoter reporter construct (pGL3-BCO1022) showing the location of putative transcription factor binding sites. *Below*, deletion analysis of the BCMO1 -987/+35 promoter fragment. TC-7 cells were transiently transfected with luciferase reporter constructs containing serial deletions of BCMO1 5'-flanking DNA. Cells were transfected with reporter constructs (0.3 μg/well) and a β-Gal expression vector was added as an internal control. After 24 h, cells were lysed and luciferase and β-Gal activities were measured. Results are means ± S.D. of three or more independent experiments each performed in triplicate. *, *p *< 0.05, **, *p *< 0.001.

To evaluate the functionality of these potential *cis*-acting elements, the ~1.0 kb nucleotide sequence upstream from the human BCMO1 coding sequence start site was excised from pBCO1022-CR (Additional File 1, A) and ligated into the pGL3-basic luciferase reporter vector (pGL3-BCO1022). To determine whether this sequence confers promoter activity, several cell lines including TC-7 were transiently transfected with the pGL3-BCO1022 reporter (data not shown). The human intestinal CaCo-2 derived TC-7 cell line is unusual in that it has significant endogenous BCMO1 activity [26]. We also have determined by RT-PCR that the parent CaCo-2 cell line expressed BCMO1 mRNA, although at very low levels (data not shown). Figure 1A shows this cloned DNA fragment drives enhanced luciferase expression in TC-7 cells.

To map the region in the BCMO1 gene that influences expression of the luciferase reporter construct, the ~1.0 kb promoter sequence was progressively deleted from the 5'-end by nested PCR to generate the deletion clones pGL3-BCO682, pGL3-BCO328, pGL3-BCO218, pGL3-BCO147 and pGL3-BCO59. Each construct was transfected into TC-7 cells. The expression vector pCMV-β-Gal was used as an internal control for adjusting transfection efficiency. As shown in Fig. 1B, deletion of the 5'-flanking 340 bp (-647/+35 promoter fragment) minimally changed luciferase activity compared to the full-length, -987/+35 genomic fragment. Further deletions of the 5'-flanking sequence (-293/+35 and -197/+35) progressively decreased luciferase activity by 25-30%. Deletion of an additional 5'-flanking 85 bp (-112/+35 promoter sequence) that includes putative MEF2, C/EBP and IRF1 sites, dramatically decreased reporter gene expression. Further deletion completely abolished BCMO1 reporter activity. As a negative control, TC-7 cells transfected with empty vector (pGL3-basic) showed no significant luciferase activity. Transfection of the minimal promoter fragment (pGL3-BCO147) containing the PPAR site (-55/-43) resulted in an approximate 12-fold induction of luciferase activity compared with the pGL3-basic vector (Figure 1B).

### MEF2 and PPAR sites in the BCMO1 promoter and basal transcription

**Figure 2:**
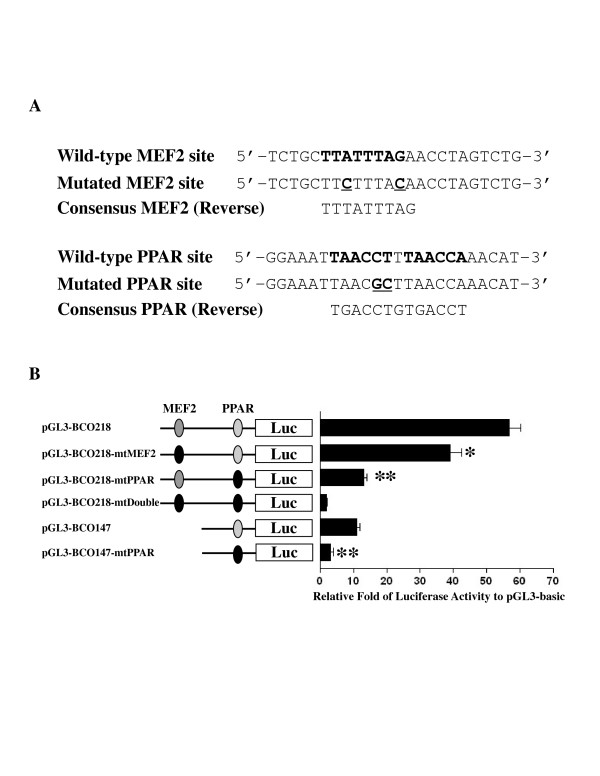
**Functional analysis of the BCMO1 promoter mutated regulatory sequences. A. **Comparison of wild type and mutant sequences for the BCMO1 promoter MEF2 binding site (-187/-163) and PPAR binding site (-61/-37). Mutated base pairs are underlined and *in bold*. **B. **Site-directed mutations (*in solid black*) were introduced into the binding sites of both MEF2 and PPAR transcription factors. TC-7 cells were transfected with 0.3 μg/well of luciferase reporter constructs containing BCMO1 proximal promoter fragments of 218 bp or 147 bp in length or with the corresponding mutated sequences. Data are expressed as a relative fold luciferase activity compared to that of the empty vector. Results shown are means ± S.D. of three or more independent experiments each performed in triplicate. *, *p *< 0.05, **, *p *< 0.001.

The ~200 bp genomic DNA region proximal to the transcriptional start site was further investigated to determine which specific *cis-*acting elements confer basal expression of the human BCMO1 gene. Sequence analysis suggested the presence of a MEF2 binding site (TGCTTATTTAGA) (Additional file 1, A) that is absent in the mouse promoter, and a PPAR/RXR binding site (TAACCT**T**TAACCA) conserved in the mouse promoter. Therefore, the MEF2 site was mutated (Figure 2A) to test its contribution to basal transcriptional activity. As shown in Figure 2B, transfection of a reporter construct containing the mutated MEF2 binding site resulted in an approximately 30% reduction in luciferase activity compared to the wild type construct, pGL3-BCO218. A much greater reduction in reporter gene activities (pGL3-BCO218 and pGL3-BCO147) resulted from mutation of the PPAR site lying within the proximal promoter sequence. Mutation of both MEF2 and PPAR sites within the proximal promoter region of BCMO1 (pGL3-BCO218) abrogated the expression of reporter gene.

### Verification of MEF2 and PPAR binding to the BCMO1 promoter

The following experiments were undertaken to verify that endogenous transcription factors bind to these different BCMO1 proximal promoter response elements. As shown in the Western blots reproduced in Additional file 2A, TC-7 cell nuclear extracts contain PPAR isoforms (PPARα, β and γ), RXRα, RARβ and MEF2 isoforms (MEF2A, 2C and 2D). The capacity of MEF2 to bind the corresponding BCMO1 elements was then tested using EMSA with TC-7 nuclear protein extracts and radiolabeled probes corresponding to the wild type or mutated MEF2 binding sequence (nt -188 to -165) (additional file 2B). A MEF2 DNA-protein complex was detected (Additional File 2C, *left panel*) having electrophoretic mobilities corresponding to heterodimeric or homodimeric MEF2 isoforms, consistent with previously reported observations [27]. Specificity of MEF2 binding was verified by three criteria. First, specific DNA-protein binding was eliminated by the addition of 100-fold molar excess of the non-radiolabeled specific oligonucleotide. Second, substitution of radiolabeled oligonucleotide in which the MEF2 site was mutated also abolished DNA-protein complex formation (Additional File 2C, *left panel*). Finally, to further assess the identity of TC-7 cell nuclear proteins binding to the MEF2 site, supershift analysis was performed using specific MEF2 antibodies. The radiolabeled probe corresponding to the MEF2 binding site was supershifted by addition of an antibody to MEF2C to an extent that corresponded to its abundance in TC-7 cell nuclei (Additional file 2A). Supershift assays showed diminished intensity of DNA-protein complex with antibody against MEF2A and an appearance of a supershifted band with antibody against MEF2D. (Additional file 2C, *right panel*).

Interrogation of the BCMO1 promoter PPAR-response element (PPRE) (-60/-37) using EMSA yielded a single PPRE DNA-protein band with the expected mobility (additional file 2D, *left panel*). Similar to the MEF2 EMSA, this binding was specific, as it was inhibited by addition of excess cold specific oligonucleotide and abolished by substitution of a mutated PPRE oligonucleotide probe (additional file 2B and 2D, *left panel*). The specificity of this interaction was further observed by the supershift assay. Binding of members of the PPAR family of transcription factors, RXR and RAR transcription factors was demonstrated using the PPAR site (-60/-37) as a probe. The results showed diminished intensity of the bound lower band (relative to the upper band) with addition of antibodies against PPARγ and RXRα as well as the appearance of a weakly detectable supershifted band with addition of RXRα antibody (Additional file 2D, *right panel*). The extent of supershift with PPARγ antibody corresponded to its low abundance in TC-7 cell nuclei as shown in Additional File 2A.

The region of the human BCMO1 promoter flanked by the MEF2 (-185/-173) and AP2 (-69/-61) elements contains a cluster of potential regulatory elements. A comparison of this DNA region to the BCMO1 5'-flanking regions in other currently sequenced genomes shows the MEF2 site (-185/-173) and a putative C/EBP site (-165/-155) are uniquely present in the human BCMO1 promoter (data not shown). The C/EBP DNA response element showed specific transcription factor binding in TC-7 cells, but DNA-protein binding did not significantly alter transcriptional activity of the promoter reporter constructs (data not shown). Putative IRF-1 and GATA1 binding sites in this region are represented both in the aligned human and mouse BCMO1 promoters. However, no specific protein binding to either of these DNA elements in the TC-7 cell system was detected (data not shown).

### Cooperation of MEF2C and PPARγ/RXRα in intestinal BCMO1 promoter activity

As an alternative strategy for characterizing the dependence of BCMO1 transcription on MEF2 and PPAR isoforms, in various experiments we co-transfected a proximal BCMO1 reporter construct (pGL3-BCO218) with mammalian expression vectors for MEF2C, PPARγ, RXRα or PPARγ /RXRα, each of the latter under the control of a cytomegalovirus or Rous sarcoma virus promoter. Relative luciferase activity was normalized to that resulting from co-transfection with the empty vector, pcDNA3. Cells in which MEF2C was over-expressed showed an approximately three-fold increase in relative luciferase activity. Conversely, expression of a dominant negative MEF2 (MEF2A-131) reduced activity of the BCMO1 promoter reporter gene by half (Additional File 3A). As shown in Additional File 3B, approximately 1.5, 1.4 and three-fold increases in pGL3-BCO218 luciferase activity were induced by PPARγ, RXRα and PPARγ /RXRα, respectively. Surprisingly, co-expression of MEF2C plus PPARγ /RXRα resulted in a six-fold stimulation of the BCMO1 reporter expression (Additional File 3B), indicating MEF2 and PPARγ have an additive effect on BCMO1 promoter activity.

The role of the PPRE in the BCMO1 promoter sequence was further probed using co-expression of PPARγ /RXRα with the reporter construct containing the BCMO1 minimal promoter sequence (pGL3-BCO147). This DNA fragment contains the PPRE but lacks the MEF2 sites. The resulting reporter gene activity was dramatically amplified. As a control condition, co-expression of PPARγ /RXRα with this BCMO1 minimal promoter sequence in which the PPRE was specifically mutated resulted in no increase in luciferase activity (Additional File 3C).

To confirm the additive effects of MEF2C and PPARγ in BCMO1 promoter activation, we utilized BCMO1 promoter constructs in which the respective response elements were mutated. Wild type and mutated promoter constructs were then co-transfected into TC-7 cells with MEF2C or PPARγ /RXRα alone or in combination. As shown in Additional File 3D, the wild type proximal promoter (pGL3-BCO218) enhanced BCMO1 reporter activity when co-transfected with MEF2C, PPARγ /RXRα or the combination of MEF2C and PPARγ /RXRα. Mutation of the MEF2 site (pGL3-BCO218-mutMEF2), but not of the PPAR site, significantly reduced this enhanced BCMO1 reporter expression by 24–30%. Conversely, mutation of the PPRE (pGL3-BCO218-mutPPAR) not only dramatically decreased basal BCMO1 promoter activity, but also decreased the MEF2C and PPARγ-induced activation. The combinatorial effects of MEF2C plus PPARγ /RXRα on the BCMO1 reporter gene were abolished when either the MEF2 site or PPAR site were mutated.

## Discussion

Although the *in vivo *enzymatic reaction first was described in 1930 by Moore [28], identification of β-carotene oxygenase activity was only demonstrated in 1965 when Olsen and Hayaishi [11] and Goodman and Huang [12] independently showed rat small intestine homogenates enzymatically cleave β-carotene at the 15,15'-carbon double bond to yield two molecules of vitamin A aldehyde (retinal). More recently, this central cleavage enzyme, BCMO1, was purified [29,30], mouse [31-33] and human [34] cDNAs were identified and the human recombinant enzyme was biochemically characterized [35] as a monooxygenase [36].

Cleavage of β-carotene has been shown to be a source of target tissue retinoic acid production in the small intestine, liver, kidney, lung and testis [37]. In addition to considerable apparent tissue-specific regulation [[20], unpublished data], BCMO1 is subject to species differences in the efficiency of intestinal β-carotene to retinoid cleavage. The goal of the current studies was to identify the basal promoter and core transcriptional elements responsible for regulating human BCMO1 expression in intestinal cells. The use of CaCo-2 derived TC-7 cells was prompted by their demonstrated endogenous BCMO1 activity. TC-7 cells have a phenotype even closer to small intestine enterocytes than does the parental population gauged by expression of several additional differentiation-associated proteins and nutrient absorption patterns [38,39].

The comparison of the human and mouse promoter sequences by ClustalW and manual inspection revealed little interspecies homology. Whether this finding is relevant to the marked species differences in carotenoid absorption and metabolism is not known. In humans, although the majority of absorbed β-carotene can be converted in the intestine directly to retinal [17,18,40], considerable β-carotene levels are detected in blood. In rodent small intestine, nearly all β-carotene is directly cleaved to retinal, leaving little intact β-carotene in the circulation.

Recent data emphasize the concept that interspecies expression differences, especially in structural genes such as enzymes, are less the result of select *trans*-regulatory changes with widespread effects, but rather of many *cis*-acting changes spread throughout the genome [41,42]. The sequence context of the genomic DNA regions that contain protein-binding sites may determine whether these regions function in transcriptional regulation. Since closely spaced transcription factor binding sites can facilitate protein-protein interactions, clustering of protein-binding elements is often a hallmark of a subset of the control regions in genomic DNA [43-46].

We found the proximal 700 bp of genomic 5'-flanking sequence conferred maximal BCMO1 promoter activity in a homologous human intestinal cell system. Our functional studies (promoter deletion experiments, site-directed mutagenesis assays, EMSA, supershift, ability to drive expression of reporter genes) establish that the minimal region of the human BCMO1 promoter required for induction of the gene is located within 200 bp upstream from the start site of transcription, a region that contains a cluster of *cis*-acting elements. Mutation of the PPRE or MEF2 binding sites reduced basal promoter activity. Mutation of both promoter elements abrogated BCMO1 transcription in intact cells, a result additive of the effects of mutating either site singly. Coupled with the direct experimental confirmation of specific binding, the PPRE and the human-specific MEF2 site together regulate basal BCMO1 expression. This result differs from the mouse, in which the PPRE solely is both necessary and sufficient for the restricted expression of BCMO1 [21].

The MEF2 family of transcription factors was initially identified from muscle cells due to binding to an A/T rich consensus sequence [c/tTA(A/T_4_)TAg/a] found in the regulatory region of many muscle specific genes [47]. It since has become apparent that MEF2 transcription factors participate in diverse gene regulatory programs, including those for muscle and neural differentiation, cardiac morphogenesis and blood vessel formation [48]. Four mammalian isoforms of MEF2 (A to D), encoded by separate genes, have been identified [47]. However, although MEF2 has been shown to play important roles in several cell types including skeletal muscle, neurons, T cells and other non-muscle cells [48-50], to our knowledge, a role for MEF2 has not previously been described in intestinal epithelial cells. In the present work, we provide evidence that three isoforms of MEF2 (MEF2A, C and D) are expressed in intestinal TC-7 cells (Additional file 2A). Isoforms of MEF2 have been studied in several biological systems, including muscle, neurons, and immune cells, where multiple isoforms are present [48]. Whether different isoforms of MEF2 present in the same cell may perform distinct molecular functions remains largely unknown. Complexity arises from the observations that different isoforms of MEF2 can form either heterodimers or homodimers having apparently indistinguishable DNA-binding specificity. Our data show MEF2C is the major MEF2 isoform that binds to BCMO1 promoter. Consistent with this observation, our data showed that over-expression of MEF2C significantly transactivated BCMO1 reporter gene activity in TC-7 cells.

MEF2 proteins interact with and potentiate the action of other classes of lineage-specific transcription factors. In the present study, we also demonstrated that MEF2C and PPARγ cooperatively regulate BCMO1 gene expression, an observation reminiscent of the interaction between MEF2 proteins and myogenic bHLH factors in skeletal muscles. Several studies have reported functional interaction between MEF2 proteins and members of the nuclear receptor superfamily, such as MEF2A and thyroid hormone receptor (TR) synergism to activate α-cardiac MHC gene expression [51] and MEF2C and PPARα cooperation to induce human carnitine palmitoyltransferase 1β (CTB1β) gene activation [52]. Given the co-expression of MEF2 and PPAR factors in several cell types including intestinal epithelial cells, the PPAR-dependent MEF2 pathway described in this work may provide a molecular paradigm for understanding the mechanism of action of MEF2 in many target cells.

Changes in expression magnitude and relative expression of genes can be a governing mechanism driving species diversification [42] via adaptation to ecological, including nutritional, niches. The importance of BCMO1 gene expression in the maintenance of vitamin A sufficiency makes the BCMO1 promoter a likely target for natural selection. Interspecies differences in efficiency of β-carotene metabolism raise the question whether different regions of the BCMO1 promoter have evolved under heterogeneous dietary constraints. The limitations of the present study preclude associating the human specific regulation of BCMO1 with specific metabolic consequences. Nevertheless, our data suggest physiological hypothesis as only certain DNA sequences functional for the binding of activators are conserved.

Further investigation should advance the understanding of transcriptional control of the key step in β-carotene to vitamin A conversion and, consequently, may have relevance for physiology and human health.

## Conclusion

We showed that the proximal ~200 bp of BCMO1 promoter region is essential for basal promoter activity of human BCMO1 in intestinal TC-7 cells. PPARγ is essential but not sufficient to activate human BCMO1 gene expression. BCMO1 expression is dependent on the cooperation between PPARγ and MEF2 isoforms. An understanding of the transcription factors and cis-acting elements involved in regulation of the human BCMO1 expression should facilitate a better understanding of the regulation of BCMO1 expression in physiologic and pathologic states of Vitamin A formation.

## Methods

### Cell line, reagents and plasmids

Clone TC-7 [53] of the human intestinal Ca Co-2 parent cell line was obtained from Dr. Alexandrine During, USDA Human Nutrition Research Center, Beltsville, MD. The PPARγ agonist (GW1929) and antagonist (GW9662), PPARα agonist (WY14643) and antagonist MK886, and PPARβ agonist (GW501516) were obtained from Alexis Biochemicals (San Diego, CA). The PPARα, PPARβ and PPARγ expression vectors were the gifts of Dr. D.P. Kelly (Washington University School of Medicine, St. Louis, Mo.). The RXRα, RARβ and β-galactosidase (β-Gal) expression vectors were gifts of Dr. P. Lefebvre (Ligue Nationale Contre le Cancer, Paris, France). The MEF2C and MEF2A-131 (dominant negative form of MEF2, DnMEF2) expression vectors were gifts from Dr. Zixu Mao (Brown Medical School, Providence, R.I.).

### Cloning of the 5'-flanking region of the human BCMO1 gene

A 1022 bp BCMO1 promoter fragment spanning nt 79828775 to 79829795 [Ensembl Gene, ENSG00000135697] [54] was amplified by the polymerase chain reaction (PCR) from human liver genomic DNA (BioChain Institute, Hayward, CA) using a 5' primer, 5'-GAATTTCAGGCAATGGCAAC-3' (corresponding to genomic DNA sequence nt 79828775 to 79828795) and a 3' primer, 5'-ACTTGTCCCTCTCCAAGAGC-3' (corresponding to nt 79829775 to 79829795). The PCR product was gel purified and ligated into pCR2.1 using a TA Cloning Kit (Invitrogen Corp., Carlsbad, CA) to create pBCO1022-CR. Orientation was confirmed by DNA sequence analysis.

### Construction of luciferase plasmids

The plasmid pGL3-basic (Promega Corp.) was used to construct pGL3-BCO1-Luc plasmids containing BCMO1 promoter fragments. pGL3-BCO1(-987/+35)-Luc, containing the DNA 5'-flanking sequence nt -987 to +35 (relative to the transcription start site [+1]), was excised from pBCO1022-CR by double digestion with *KpnI *and *XhoI*, ligated into the *KpnI*/*XhoI *sites of pGL3-Basic using T4 DNA ligase and sequenced to verify orientation. This full-length reporter plasmid was designated pGL3-BCO1022. To generate progressive 5'-unidirectional deletion mutants by PCR, a series of forward primers was used in combination with the same reverse primer and pGL3-BCO1022 as template. The reverse primer (with the *XhoI *site underlined) was 5'-CCGCTCGAGGGTGCCGAGGGAGATC-3'. The forward primers, each of which included the *KpnI *restriction site (underlined), began with: -647 (5'-CGGGGTACCGGTCTCGAACTCCTG); -293 (5'-CGGGGTACCTAATTCCCAGCACCTC); -197 (5'-CGGGGTACCGGAATTCTCTCTGC); -112 (5'CGGGGTACCAAAGCTGAGGGC); and -24 (5'-CGGGGTACCAGCGCAGCTTCCCTTG). The PCR products were gel-purified, digested with *KpnI *and *XhoI*, and subcloned into the *KpnI/XhoI *sites of pGL3-Basic. They were designated pGL3-BCO682, pGL3-BCO328, pGL3-BCO218, pGL3-BCO147 and pGL3-BCO59, respectively. Each construct was verified by sequencing the insert and plasmid flanking region.

### Site-directed mutagenesis

To generate site-directed mutants of the cAMP response element-binding protein (CREB), RAR, MEF2, CCAAT/enhancer binding protein (C/EBP) and PPAR DNA elements that are located from nt -647 to -56, the QuickChange site-directed mutagenesis kit (Stratagene, La Jolla, CA) was used according to the manufacturer's instructions. The oligonucleotides used to create the mutations are listed in Table 1, with the mutated nucleotides *in bold *and *underlined *throughout (The complementary strand sequences are not shown). Mutation of each binding sequence was confirmed by DNA sequencing.

### Transient transfection and luciferase assays

The TC-7 subline of CaCo-2 cells was cultured in Dulbecco's Modified Eagle's Medium (DMEM, Life Technologies Inc., Gaithersburg, Md.) supplemented with penicillin (100 units/ml), streptomycin (100 μg/ml) (Life Technologies), and 20% fetal bovine serum (HyClone, Logan, Ut.) at 37°C in a humidified atmosphere of 95% air and 5% CO_2_. Cells were plated in 12-well tissue culture dishes at a density of 5 × 10^4 ^cells/well for 24 h before transfection. At 70–90% confluence, cells were transfected with 0.3 μg of the indicated constructs using 1 μl of LipofectAMINE 2000 transfection reagent per well mixed in Opti-MEM I (Invitrogen). Co-transfection with a β-galactosidase expression plasmid was used to determine efficiency of each transfection. For the co-transfection assays, the total amount of DNA for each transfection was kept constant by using a control vector (pcDNA3). At various time points, cell lysates were analyzed for luciferase and β-galactosidase activities. Luciferase activity relative to the pGL3-basic transfectants was determined after adjustment for β-galactosidase level. All transfections were performed in triplicate for at least three independent experiments.

### Electrophoretic mobility shift assay (EMSA)and supershift assays

Nuclear extracts from TC-7 cells were prepared as described previously [21]. Double-stranded oligonucleotides containing sequences from the human BCMO1 promoter (shown in Table 2) were synthesized (Operon Biotechnologies, Inc., Huntsville, Ala.). EMSA reaction mixtures (20 μl final volumes) included 5–15 μg of nuclear extract, 25 mM HEPES, 100 mM KCl, 0.1% Nonidet P-40 (v/v), 1 mM dithiothreitol, 5% glycerol and 1 μg of poly(dI-dC) (Sigma, St. Louis, Mo.) as a nonspecific competitor. After incubation for 20 min on ice, 20 fmol of [γ-^32^P]-ATP end-labeled probe was added and the reaction was incubated for 30 min at room temperature. Protein-DNA complexes were separated on a 5% non-denaturing polyacrylamide gel in 0.5 × Tris-borate-EDTA buffer at 25°C. For competition assays, 100-fold molar excess of unlabeled double-stranded oligonucleotide competitor was incubated together with the nuclear extract prior to adding the ^32^P-labeled probe. For supershift EMSA, antibodies were preincubated with the nuclear extract mixtures in the binding reaction for 20 min before the probe was added.

### Western blot analysis

TC-7 cells were harvested, lysed in 1 ml of phosphate-buffered saline (PBS) containing 1% Triton X-100, 0.1% SDS, 1 mM dithiothreitol and 1 mM phenylmethylsulfonyl fluoride and centrifuged at 1,500 × *g *for 5 min. Protein concentrations were determined by the BCA protein assay (Pierce Biotechnology, Inc., Rockford, Il.), and 40 μg cell lysate samples were fractionated by SDS-PAGE. Proteins were transferred to Immobilon-P membranes (Millipore, Billerica, MA) by semi-dry blotting. The membrane was treated according to a standard Western Blotting protocol with chemiluminiscence detection. Rabbit polyclonal MEF2C (Cell Signaling Technology, Inc., Beverly, Mass.), MEF2D (BD Transduction Laboratories, Lexington, Ky.), MEF2A, PPARα, PPARβ, PPARγ, RARα and RXRα (Santa Cruz Biotechnology, Santa Cruz, CA) antibodies were diluted to 1:500 to 1:1000 in PBS, 5% non-fat dry milk. Protein-antibody interactions were detected by enhanced chemiluminescence (Amersham Biosciences Corp., Piscataway, NJ).

### Statistics

Each experiment was performed in triplicate, and the reporter values represent the mean of three to five separate cultures ± standard deviation. The significance of the difference was determined using Student's *t *test.

## Abbreviations

BCMO1, β, β-carotene 15, 15'-monooxygenase 1; RA, retinoic acid; RAR, retinoic acid receptor; RXR, retinoid X receptor; C/EBP, CCAAT/enhancer-binding protein; MEF2, myocyte enhancer factor 2; PPAR, peroxisome proliferator-activated receptor; PPRE, PPAR-response element; EMSA, electrophoretic mobility shift assay;

## Authors' contributions

X.G.: performance and interpretation of all experiments and manuscript preparation; S.T.: participation in the planning of the study; B.Y.: support to X.G.s' experiments; L.P.R.: conceived the study, design and coordination of the study, manuscript preparation. All authors read and approved the final version of the manuscript.

## Supplementary Material

Additional file 1 **A**. Nucleotide sequence of the human β, β-carotene 15,15’-monooxygenase 1 (BCMO1) promoter. Nucleotides are numbered relative to the transcriptional start site (+1) [34]. Potential consensus sequences for transcription factor binding sites are underlined and labeled. The sequence was analyzed according to the TESS program and TRACSFAC transcription factor database.
**B**. Alignment of ~900-bp of human and mouse proximal BCMO1 genomic sequence by the ClustalW (1.82) program [55]. The numbering is relative to the transcription initiation site (+1). The sequences in bold and labeled indicate putative DNA regulatory elements common to the two promoters. Click here for file

Additional file 2 **A**. Profile of RARβ, RXRα, MEF2 and PPAR transcription factors expressed in TC-7 cells. Total protein was extracted from TC-7 cells and specific immunoreactive proteins were determined by western blotting using the indicated antibodies and methods described in “Methods”.
**B**. The oligonucleotides, each matching the human BCMO1 promoter wild type or mutated MEF2 site or PPAR site. Mutated base pairs are underlined.
**C**. MEF2 proteins binding to sequence in the BCMO1 promoter described in Panel B, was characterized by EMSA. Oligomers were end-labeled with [γ-32P]-ATP and incubated with nuclear extracts from TC-7 cells (see “Methods”). Addition of 100-fold molar excess of unlabeled competitor oligomers or mutated probes is indicated above each lane (left panel). Supershift analyses (right panel) were performed by preincubating nuclear extracts from TC-7 cells with anti-MEF2A, MEF2C and MEF2D antibodies. Arrowheads indicate specific complexes and the SS indicates supershifted bands.
**D**. PPAR proteins binding to sequence in the BCMO1 promoter described in Panel B, was characterized by EMSA. Oligomers were end-labeled with [γ-32P]-ATP and incubated with nuclear extracts from TC-7 cells (see “Methods”). Addition of 100-fold molar excess of unlabeled competitor oligomers or mutated probes is indicated above each lane (left panel). Supershift analyses (right panel) were performed by preincubating nuclear extracts from TC-7 cells with anti-PPARα, PPARγ, PPARδ, RXRα and RARβ antibodies. Arrowheads indicate specific complexes and SS indicates supershifted bands. Click here for file

Additional file 3 **A**. Effects of the empty expression vector (pcDNA3), MEF2C and the dominant negative MEF2A-131 (DnMEF2) on BCMO1 promoter activity. TC-7 cells were transiently transfected as indicated. Luciferase and β-Gal activities were measured 24 hours after transfection and relative fold of luciferase activity (compared to pGL3-Basic) was determined after adjustment for β-Gal activity. Results are presented as means ± S.D. of three independent experiments each performed in triplicate. *, *p*< 0.05, **, *p*< 0.001. **B**. MEF2C and PPARγ/RXRα activate the BCMO1 gene promoter. TC-7 cells were transiently transfected with the pGL3-BCO218 reporter vector in the absence or presence of MEF2C, PPARγ, RXRα or empty expression vector, pcDNA3, as indicated. Luciferase and β-Gal activity were measured 24 hours after transfection and relative fold luciferase activity was determined after adjusting for β-Gal activity. Results are presented as means ± S.D. of three independent experiments each performed in triplicate. *, *p*< 0.05, **, *p*< 0.001. **C**. Effects of PPARγ/RXRα on BCMO1 promoter activity. TC-7 cells were transiently transfected with the native (pGL3-BCO147) or PPAR-mutated (pGL3-BCO147-mtPPAR) BCMO1 minimal promoter constructs. Co-transfections were preformed with the empty mammalian expression vector pcDNA3 or with vector containing PPARγ and RXRα, as indicated. Luciferase and β-Gal activity were measured 24 hours after transfection and relative fold luciferase activity was determined after adjusting for β-Gal activity. Results are presented as means ± S.D. of three independent experiments each performed in triplicate. **, *p*< 0.001. **D**. Mutation of MEF2 and PPAR DNA binding sites prevents BCMO1 promoter activation by MEF2C and PPARγ/RXRα. TC-7 cells were transiently transfected with 0.3 μg/well of wild-type (pGL3-BCO218) or mutant (pGL3-BCO218-mtPPAR, pGL3-BCO218-mtMEF2) reporter constructs and with expression vectors for MEF2C or PPARγ/RXRα alone or in combination. Luciferase and β-Gal activity were measured 24 hours after transfection and relative fold of luciferase activity was determined after adjusting for β-Gal activity. Results are presented as means ± S.D. of three independent experiments each performed in triplicate. All results are significantly decreased compared with wild-type reporter activity. *,  *p*< 0.05, **,  *p*< 0.001. Click here for file

## Figures and Tables

**Table 1:** Oligonucleotides used in PCR-based site-directed mutagenesis

Primer sequence	Mutated site^*a*^
5'-GGAAATTAA**CGC**TTAACCAAAC-3'	PPAR/RXR -52/-50
5'-TAGTCTGAA**AGT**AGCTTTT-3'	C/EBP -161/-159
5'-TCTGCTT**C**TTTA**C**AACCTAGTCTG-3'	MEF2 -179/-174
5'-ACTCCTG**TG**CTCAAGTG**TG**CCG-3'	CRE/RAR -633/-623

^*a *^Initiation site for BCMO1 transcription, +1. The mutated nucleotides are *in bold*.

**Table 2:** Oligonucleotides used for EMSA for putative binding sites

Oligonucleotide sequence^*a*^	DNA element
5'-GCTTGGAAATTAACCTTTAACCAAACAT-3'	Wild-type PPAR
5'-GCTTGGAAATTAtgCTTTAtgCAAACAT-3'	Mutated PPAR
5'-GGATTAAAGATTAGCTATGACACCATG-3'	Wild-type GATA-1
5'-GGATTAActcTTAGCTATGACACCATG-3'	Mutated GATA-1
5'-GGTTAAGTCAAAAGTGAAACTGGAG-3'	Wild-type IRF-1
5'-GGTTAAGTCAtcAGTGAAACTGGAG-3'	Mutated IRF-1
5'-CTCTCTGCTTATTTAGAACCTAGTC-3'	Wild-type MEF2
5'-CTCTCTGCTTgTTTAcAACCTAGTC-3'	Mutated MEF2
5'-GAACTCCTGACCTCAAGTGACCCGCCAC-3'	Wild-type CRE/RAR
5'-GAACTCCTGtgCTCAAGTGtgCCGCCAC-3'	Mutated CRE/RAR

^*a *^Upper strands of oligonucleotides with putative consensus sequences are *underlined; *the mutated nucleotides are in *lower case characters*.
