# Supplementary material for: Cooperation between MEF2 and PPARγ in human intestinal β,β-carotene 15,15'-monooxygenase gene expression
Source: BMC Mol Biol. 2006 Feb 21;7:7. doi: 10.1186/1471-2199-7-7 (PMC1526748; doi:10.1186/1471-2199-7-7)
Supplement: Additional file 1 — A. Nucleotide sequence of the human β, β-carotene 15,15’-monooxygenase 1 (BCMO1) promoter. Nucleotides are numbered relative to the transcriptional start site (+1) [34]. Potential consensus sequences for transcription factor binding sites are underlined and labeled. The sequence was analyzed according to the TESS program and TRACSFAC transcription factor database. B. Alignment of ~900-bp of human and mouse proximal BCMO1 genomic sequence by the ClustalW (1.82) program [55]. The numbering is relative to the transcription initiation site (+1). The sequences in bold and labeled indicate putative DNA regulatory elements common to the two promoters. [file 1471-2199-7-7-S1.pdf]

**A**

-995 CAAGTTGAAT ACGAATTTCA GGCAATGGCA

-965 ACACACGAAG ATACACGAAT AATAGGGAAG CTCAGATCAA ATTCAGGCAT TGTTTTGCCC

-905 TCTTGATAGG CCAGGAAGTT GAAGTTTCTT TTTAACTATT CTGTTTACTT ATTTATTTTT

-845 TGAGACAGAG TCTCACTCTG TGGCCGAGGC TGGAGTGCAG TGGTGCCATC TCAGCTTACT

-785 GCAATGTCTG CCTCACAGGT TCAAGCGATT TTCCTGCCTC AGCCTCCTTA GCATCTGGGA

-725 TTGCAGGCGT CTTCCACCAC GCCTGGATAA TTTTTGTATT TTTAGTAGAG ACAGGATTTC

-665 GCCGTGTTGG CCAGGCTGGT CTCGAACTCC **TGACCT**CAAG **TGACCC**GCCA ACCATGGCCT

*RAR CRE*

-605 CCCAAAGTTC TGGGATTACA GGCATGAGCC ACCACTCCTG GCCCGTCTTT TTTACTATTC

-545 TTTATAGTTT CTGAGAAAAG CCTTTGGTTC CCGTTCGTTA TTTTTTAAAT AGGTACATGA

-485 AACATTTTAT GTCTCCTTCT TCCAAAAAGA AAATAATGAA TTAAGTGGCT CTTTATGTAT

-425 GATTATTAGA GGGGAGCATC GGGGCCTTTA CTAAAAACAA AACAAAATGT AAAACCTTTC

-365 CCGTCTCTGC TGAAGGGGTT CCCTGCTGTG CAGCAGACAT TCAGTGATCA CAGAGCTTAG

-305 CTCAAGTTTC TCCTAATTCC CAGCACCTCT GATTCCCAGC ATCTCTTCTT GTTCTGATCA

-245 TAAACAACTC CAGCTCTTGG ATTACGTCGG GCAACATAAC AGCAGGCACG GAATTCTCTC

-185 **TGCTTATTTA G**AACCTAGTC **TGAAGCAAGC** TTTTTTTTTT TTTTGGTTAA G**TCAAAAGTG**

*MEF2* *C/EBP* *IRF1*

-125 AAACTGGAGA GGGAAAGCTG AGGGAACAAT AAGAGGATTA **AAGATTAGC**T ATGACA**CATG**

*GATA-1*

-065 **GCTTG**GAAAT **TAACCT**T**TAA CCA**AACATCT TATAAGTAAC GCCAGCGCAG CTTCCCTTGT

*AP2* *PPAR/RXR*

-005 GAATG**T**AAAG AGATCCAGGG CTCTTGGAGA GGGACAAGTG AGAGCCAGCC AAAAAGGAAA

*+1*

AAGCAAAGGC AGAAACGGCA TCAGGAGAGA CAGAGATGTG AAGGAGGGAA GGAGCAGGAG

AGCAGGAAGG AAACGCAGGA GGAGGGAGCA GCATCTCCTG TGAACACAGA GGAGCACCTG

TTTGCTGTTA AAATCGATCT CCCTCGGCAC CCTGAGCA**AT G**

# B

```
Human   -965 ACACACGAAGATACAC-GAATAATAGGGAAGCTCAGATCAAATTCAGGCATTGTTTTGCC
Mouse   -965 ----------CTCTACTGACTGATCCCTGTCTCCAATCCCAAAGCATCTTTAGTTTTATA
                        *  ** ** * **         **   * **  **    * *****

Human   -906 CTCTTGATAGGCCAGGAAGTTGAAGTTTCTTTTTAACTATTCTGTTTACTTATTTATTTT
Mouse   -915 ATTTCTTTAATTACATAAGACAGGGAGATCATACATATTCACGAGGTGTCCTGTGGTGTT
              * *   **       ***     *      *  *  *   *    *      *  * **

Human   -846 TTGAGACAGAGTCTCACTCTGTGGCCGAGGCTGGA-GTGCAGTGGTGCCATCTCAGCTTA
Mouse   -855 TTGATTC-----CTGTATATGTGGTGGAGTGTGGAAGCCTTCTGCTGCTCTCCTGGCTGA
             ****  *     **   * *****  ***  **** *     ** ***  **   *** *

Human   -787 CTGCAATGTCTGCCTCA-CAGGTTCAAGCGATTTTCCTGCCTCAGCCTCCTTAGCATCTG
Mouse   -800 GGGCGTCACTTCGAGCAGCAGGAACCGAGAATCCCCCAATTCCCAC--CCTATGCTCTCA
               **      *    ** ****  *     **   **     *  *  ***  **

Human   -728 GGATTGCAG---GCGTCTTCCACCACGCCTGGATAATTTTTGTATTTTTAGTAGA---GA
Mouse   -742 AAAATGCAGTGTGGATTTTAGTCTTTGGGTCCTCCCTCTTATCAGTCTGAGTTGAACTGA
               * *****   *  * **   *   *  *      * **   * * * *** **   **

Human   -674 CAGGATTTCGCCGTGTTGGCCAGGCTG---GTCTC---GAACTCCTGACCTCAAGTGACC
Mouse   -682 AGGGTTCCCGATGGACTGGCTATGCAGAAGGCCTCAGGGATCCTCCTGTCTCTGCCTCCC
               ** *  **  *   **** * ** *   * ***   ** *  *    ***      **

Human   -620 CGCCAACCATGGCCTCCCAAAGTTCTGGG----ATTACAGGCATGAGC----CACCACTC
Mouse   -622 AGCTCTGGAATTCATTCGGGTGCTCTGGGCAGAATTCAAAGCCTCGCTTGTGCACAGGCC
              **     *   * * *    * ******    ***  * ** *        ***    *

Human   -568 CTGGC-CCGTCTTTTTTACTATTC--TTTATAGTTTCTGAGAAA----AGCCTTTGG-TT
Mouse   -562 CTGACGTTGTTTTGTGTTTTAATTGTTCTTTGGTTCCCGAAAAAGCCAAGTCTTTGTCTT
             *** *   ** ** * *  ** *   * * * *** * ** ***    ** *****  **

Human   -516 CCCGTTCGTTATTTTTTAAATAGGTACATGAAACATTTTATGTCTCCTTCTTCCAAAAAG
Mouse   -502 CCCCATCTTTGCTTTTTAAATGGGCACATGACACATCTT-TGAGCCCTCCTTCCAAAAAG
             ***  ** **  ********* ** ****** **** ** **   *** ***********

Human   -456 AAAATAATGAATTAAGTGGCTCTTTATGTATGATTATTAGAGGGGAGCATCGGGGCCTTT
Mouse   -443 AAAACCA---ATCAAGTGTCCGTTGATAGGATTTTTTTTAAGAGTGGGAACGA-------
             ****  *   ** ***** *  ** **      ** **  ** *  * * **

Human   -396 ACTAAAAACAAAACAAAATGTAAAACCTTTCCCGTCTCTGCTGAAGGGGTTCCCTGCTGT
Mouse   -393 ACCAAGCCCCAGGTTAAAATCAAAGCCTTCTCTCTCTGTTTGCAGAAGGCAAAGATGTCA
             ** **   * *    ***   *** ****  *  *** *    *   **        *

Human   -336 GCAGCAGACATTCAGTGATCACAGAGCTTAGCTC---AAGTTTCTCCTAATTCCCAGCAC
Mouse   -333 CCAGCCCACAACTTGCAATAGTGCGGTCAATGTCTTTGATGGTCCCCTCGCTTGAAGAGC
              ****  ***    *  **      *   *  **    *   ** ***   *   **  *

Human   -279 CTCTGAT--TCCCA---GCATCTCTTCTTGTTCTGATCATAAACAACTCCAGCTCT----
Mouse   -273 CTTTGCTGATCCCAAATGCTGTTCTCTCTGCTCTAACCATAAATGCATCCAGTTCTCAGG
             ** ** *  *****   **   ***   ** *** * ******    ***** ***

Human   -228 -TGGATTACGTCGGGCAA-CATAACAGCAGGCACGGAATTCTCTCTGCTTATTTAGAACC
Mouse   -213 ATGGGCTAGCTCGGGCAAACTCAGCAGAACGCGCCAAATCATCTCTTCCGACTCAGAGCG
              ***  **  ******** *  * *** * ** *  ***  ***** *  * * *** *

                                                      IRF1
Human   -170 TAGTCTGAAGCAAGCTTTTTTTTTTTTTTTGGTTAAGTCAAAAGTGAAACTGGAGAGGGAA
Mouse   -153 GAGCCT------------------TCCTTGGCTAAGCTAAAAGTGAAATCAGAGCGGAGG
              ** **                  *  **** ****  **********   *** **

                                          GATA1         bHLH  AP2
Human   -110 AGCTGAGGGAACAATAAGAGGATTAAAGATTAGCTATGACACATGGCTTGGAAATTAACC
Mouse   -111 AGTTGGAAAAACACTAATGGGATTAAAGATTAGCAGTAACACATGGCTTGGGAACTGACC
             ** **    **** ***  ***************  * ************* ** * ***

             PPAR/RXR     TATA
Human    -50 TTTAACCAAACAT-CTTATAA-GTAACGCCAGCGCAGCTTCCCTTGTGAATG –1
Mouse    -51 TTTGACCTAACACACACACACTGTATAAATTACCCAGCAGCTTCCGGCAGC- -1
             *** *** ****  *  * *  ***       * ****  *    *  *
```
