# Supplementary material for: Cooperation between MEF2 and PPARγ in human intestinal β,β-carotene 15,15'-monooxygenase gene expression
Source: BMC Mol Biol. 2006 Feb 21;7:7. doi: 10.1186/1471-2199-7-7 (PMC1526748; doi:10.1186/1471-2199-7-7)
Supplement: Additional file 2 — A. Profile of RARβ, RXRα, MEF2 and PPAR transcription factors expressed in TC-7 cells. Total protein was extracted from TC-7 cells and specific immunoreactive proteins were determined by western blotting using the indicated antibodies and methods described in “Methods”. B. The oligonucleotides, each matching the human BCMO1 promoter wild type or mutated MEF2 site or PPAR site. Mutated base pairs are underlined. C. MEF2 proteins binding to sequence in the BCMO1 promoter described in Panel B, was characterized by EMSA. Oligomers were end-labeled with [γ-32P]-ATP and incubated with nuclear extracts from TC-7 cells (see “Methods”). Addition of 100-fold molar excess of unlabeled competitor oligomers or mutated probes is indicated above each lane (left panel). Supershift analyses (right panel) were performed by preincubating nuclear extracts from TC-7 cells with anti-MEF2A, MEF2C and MEF2D antibodies. Arrowheads indicate specific complexes and the SS indicates supershifted bands. D. PPAR proteins binding to sequence in the BCMO1 promoter described in Panel B, was characterized by EMSA. Oligomers were end-labeled with [γ-32P]-ATP and incubated with nuclear extracts from TC-7 cells (see “Methods”). Addition of 100-fold molar excess of unlabeled competitor oligomers or mutated probes is indicated above each lane (left panel). Supershift analyses (right panel) were performed by preincubating nuclear extracts from TC-7 cells with anti-PPARα, PPARγ, PPARδ, RXRα and RARβ antibodies. Arrowheads indicate specific complexes and SS indicates supershifted bands. [file 1471-2199-7-7-S2.pdf]

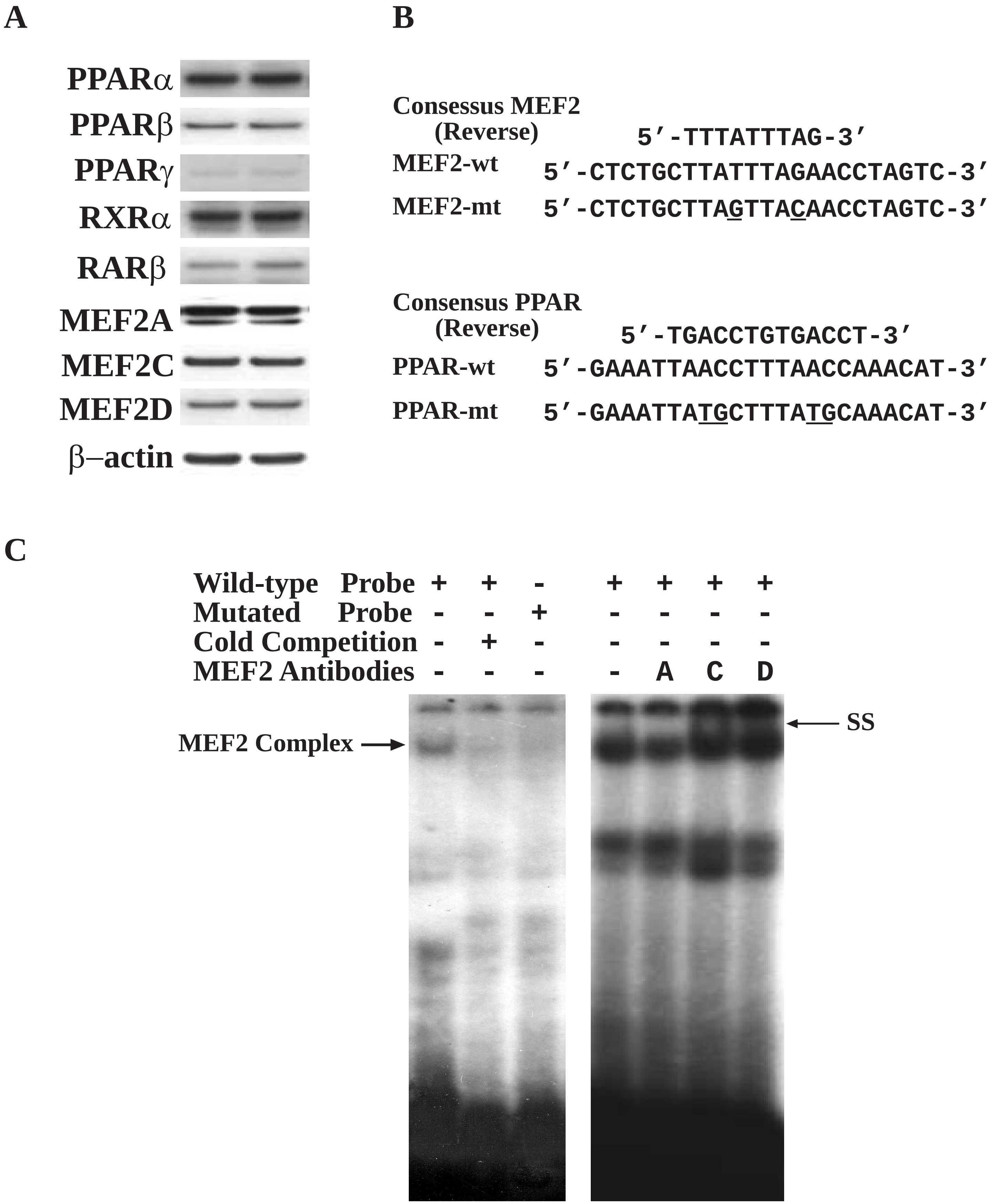
A
PPARα
PPARβ
PPARγ
RXRα
RARβ
MEF2A
MEF2C
MEF2D
β–actin
B
Consessus MEF2 (Reverse) 5′-TTTATTTAG-3′
MEF2-wt 5′-CTCTGCTTATTTAGAACCTAGTC-3′
MEF2-mt 5′-CTCTGCTTAGTTACAACCTAGTC-3′
Consensus PPAR (Reverse) 5′-TGACCTGTGACCT-3′
PPAR-wt 5′-GAAATTAACCTTTAACCAAACAT-3′
PPAR-mt 5′-GAAATTATGCTTTATGCAAACAT-3′
C
Wild-type Probe + + - + + + +
Mutated Probe - - + - - - -
Cold Competition - + - - - - -
MEF2 Antibodies - - - - A C D
MEF2 Complex
SS

D

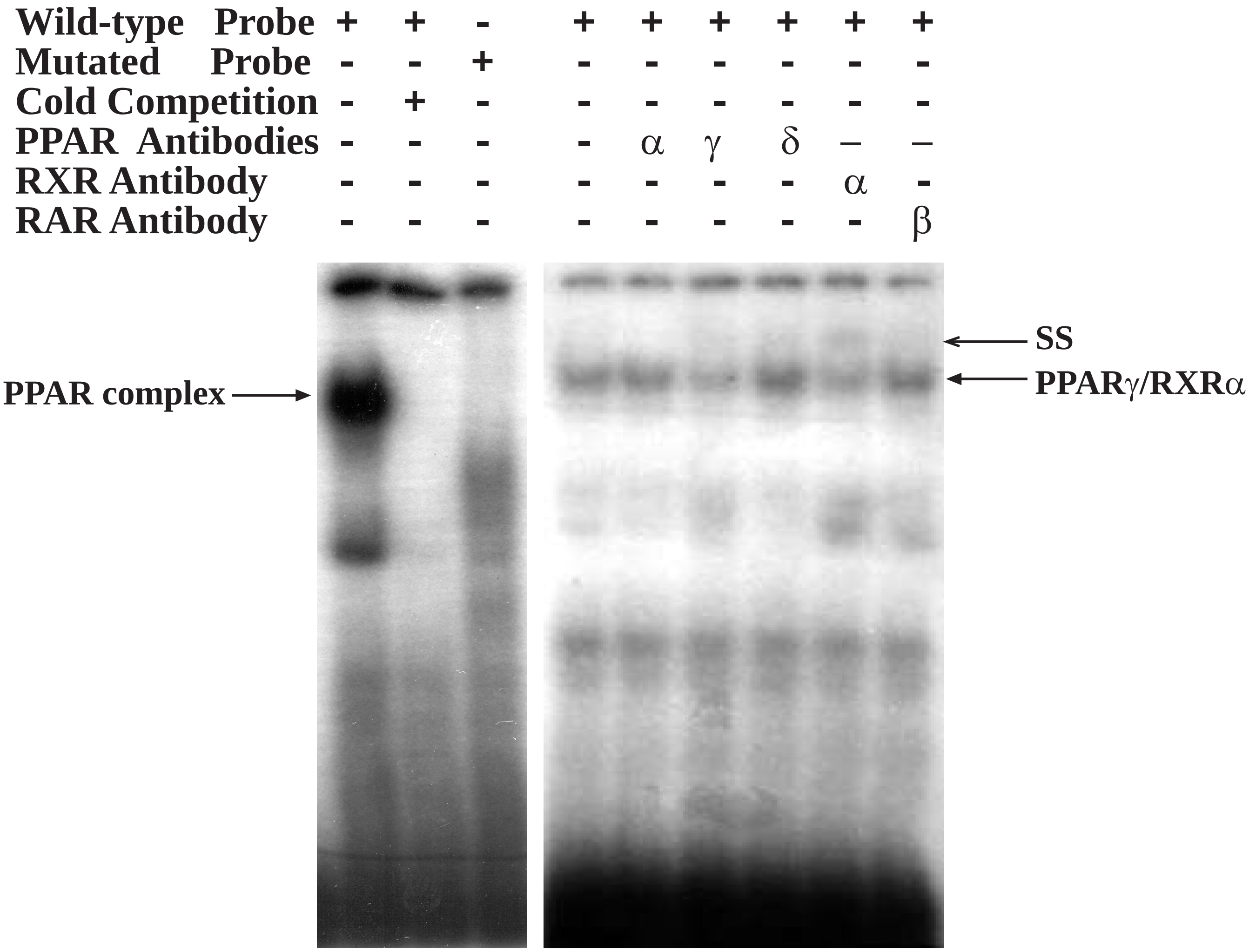

Wild-type Probe + + - + + + + + +
Mutated Probe - - + - - - - - -
Cold Competition - + - - - - - - -
PPAR Antibodies - - - - α γ δ – –
RXR Antibody - - - - - - - α -
RAR Antibody - - - - - - - - β
PPAR complex
SS
PPARγ/RXRα
