# Supplementary figures and images for: Cooperation between MEF2 and PPARγ in human intestinal β,β-carotene 15,15'-monooxygenase gene expression
Source: BMC Mol Biol. 2006 Feb 21;7:7. doi: 10.1186/1471-2199-7-7 (PMC1526748; doi:10.1186/1471-2199-7-7)

**A**

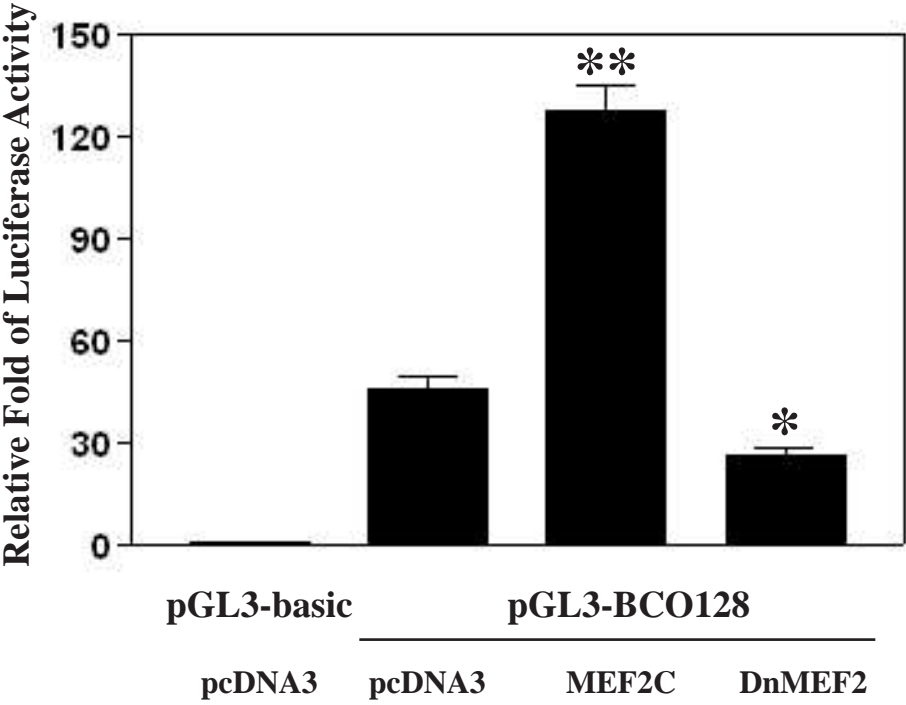

**B**

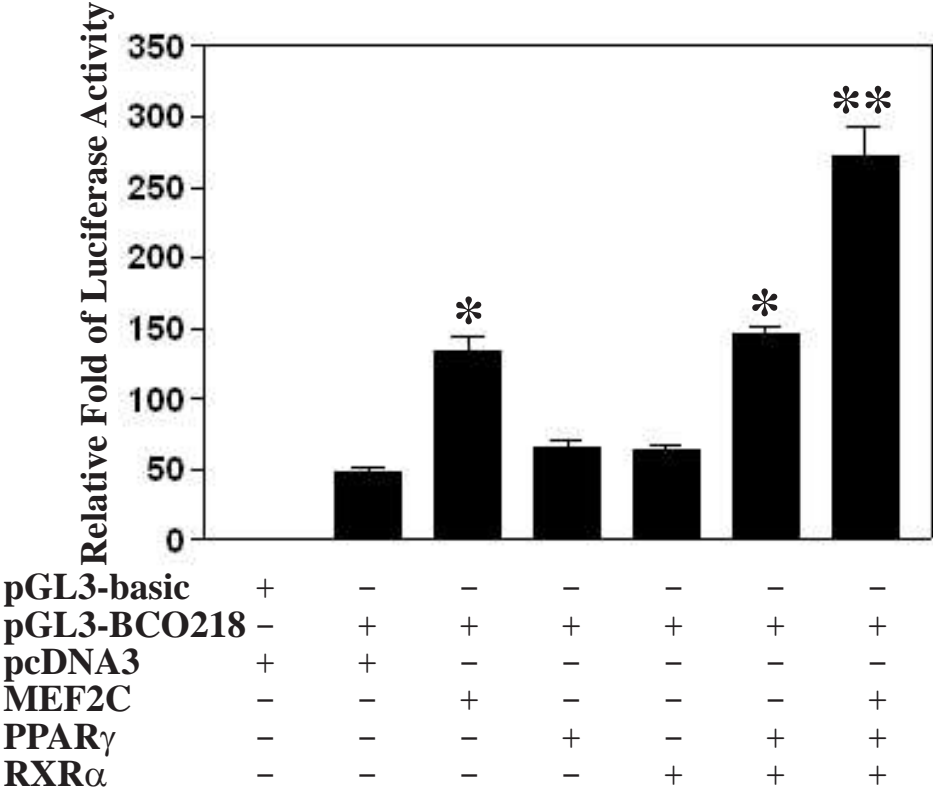

C

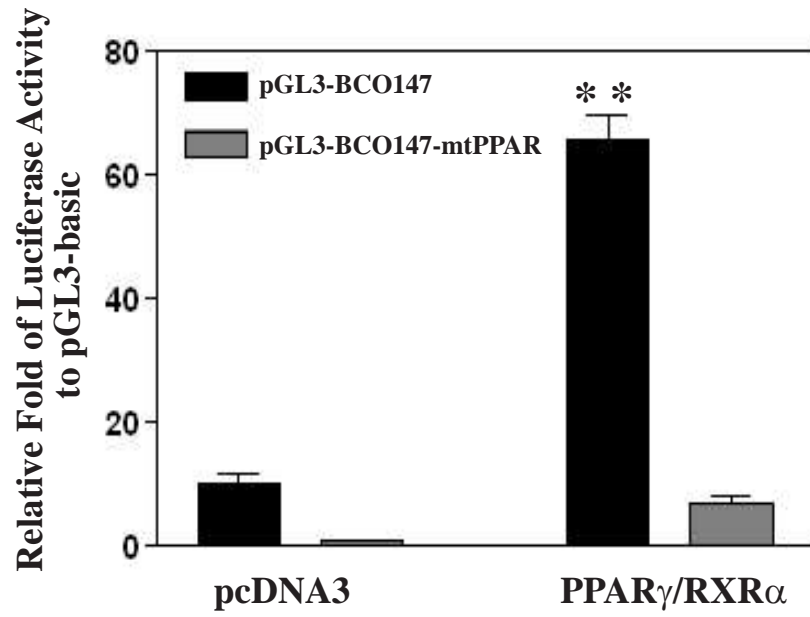

D

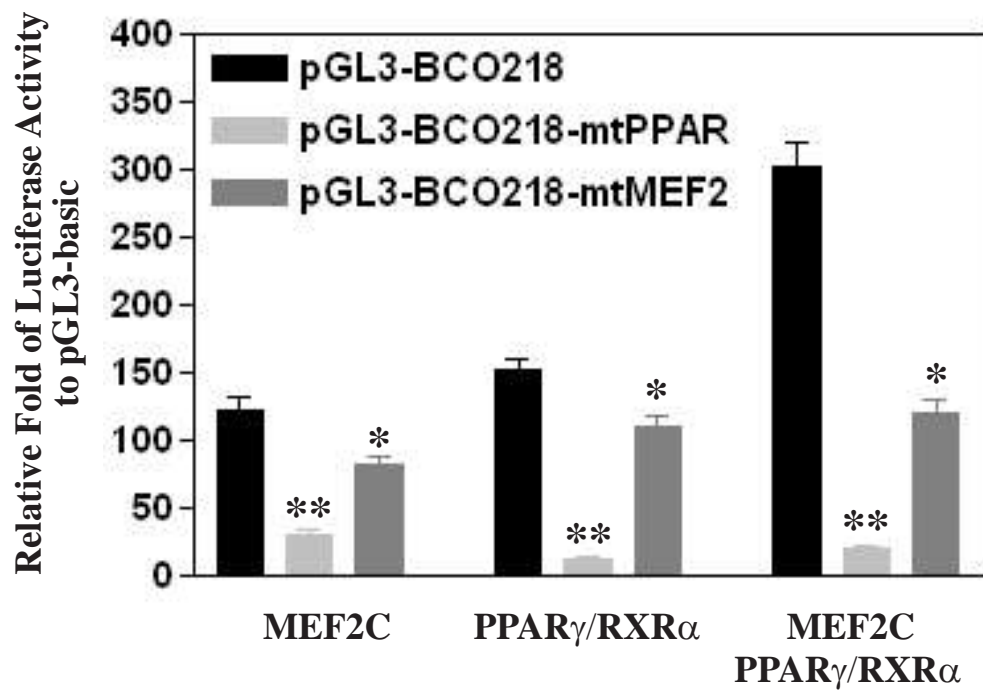

Supplement: Additional file 3 — A. Effects of the empty expression vector (pcDNA3), MEF2C and the dominant negative MEF2A-131 (DnMEF2) on BCMO1 promoter activity. TC-7 cells were transiently transfected as indicated. Luciferase and β-Gal activities were measured 24 hours after transfection and relative fold of luciferase activity (compared to pGL3-Basic) was determined after adjustment for β-Gal activity. Results are presented as means ± S.D. of three independent experiments each performed in triplicate. *, p< 0.05, **, p< 0.001. B. MEF2C and PPARγ/RXRα activate the BCMO1 gene promoter. TC-7 cells were transiently transfected with the pGL3-BCO218 reporter vector in the absence or presence of MEF2C, PPARγ, RXRα or empty expression vector, pcDNA3, as indicated. Luciferase and β-Gal activity were measured 24 hours after transfection and relative fold luciferase activity was determined after adjusting for β-Gal activity. Results are presented as means ± S.D. of three independent experiments each performed in triplicate. *, p< 0.05, **, p< 0.001. C. Effects of PPARγ/RXRα on BCMO1 promoter activity. TC-7 cells were transiently transfected with the native (pGL3-BCO147) or PPAR-mutated (pGL3-BCO147-mtPPAR) BCMO1 minimal promoter constructs. Co-transfections were preformed with the empty mammalian expression vector pcDNA3 or with vector containing PPARγ and RXRα, as indicated. Luciferase and β-Gal activity were measured 24 hours after transfection and relative fold luciferase activity was determined after adjusting for β-Gal activity. Results are presented as means ± S.D. of three independent experiments each performed in triplicate. **, p< 0.001. D. Mutation of MEF2 and PPAR DNA binding sites prevents BCMO1 promoter activation by MEF2C and PPARγ/RXRα. TC-7 cells were transiently transfected with 0.3 μg/well of wild-type (pGL3-BCO218) or mutant (pGL3-BCO218-mtPPAR, pGL3-BCO218-mtMEF2) reporter constructs and with expression vectors for MEF2C or PPARγ/RXRα alone or in combination. Luciferase and β-Gal activ [file 1471-2199-7-7-S3.pdf]
